# Lipocalin-2 Inhibits Osteosarcoma Cell Metastasis by Suppressing MET Expression via the MEK–ERK Pathway

**DOI:** 10.3390/cancers13133181

**Published:** 2021-06-25

**Authors:** Ko-Hsiu Lu, Jia-Sin Yang, Yi-Hsien Hsieh, Hsiao-Ju Chu, Chia-Hsuan Chou, Eric Wun-Hao Lu, Chiao-Wen Lin, Shun-Fa Yang

**Affiliations:** 1 Department of Orthopedics, Chung Shan Medical University Hospital, Taichung 402, Taiwan; cshy307@csh.org.tw; 2 School of Medicine, Chung Shan Medical University, Taichung 402, Taiwan; 3 Institute of Medicine, Chung Shan Medical University, Taichung 402, Taiwan; gazn_sheep@yahoo.com.tw (J.-S.Y.); hyhsien@csmu.edu.tw (Y.-H.H.); pig191919@seed.net.tw (H.-J.C.); wishwing1109@gmail.com (C.-H.C.); 4 Department of Medical Research, Chung Shan Medical University Hospital, Taichung 402, Taiwan; 5 American School in Taichung, Taichung 406, Taiwan; 21ericl@ast.tc.edu.tw; 6 Institute of Oral Sciences, Chung Shan Medical University, Taichung 402, Taiwan; 7 Department of Dentistry, Chung Shan Medical University Hospital, Taichung 402, Taiwan

**Keywords:** LCN2, metastasis, osteosarcoma, MET

## Abstract

**Simple Summary:**

Higher neutrophil-derived cytokine lipocalin-2 (LCN2) expression possesses a versatile role in a myriad of cancers, but little is known about the role of LCN2 on osteosarcoma metastasis. In this study, we demonstrated that higher LCN2 inhibited cellular motility, migration, and invasion of osteosarcoma cells. Moreover, the phosphorylation of extracellular signal-regulated kinase (ERK) 1/2 was decreased by LCN2 knockdown. Conclusively, LCN2 inhibits osteosarcoma cell metastasis by suppressing MET via the mitogen-activated protein kinases/ERK kinase (MEK)–ERK pathway.

**Abstract:**

Higher neutrophil-derived cytokine lipocalin-2 (LCN2) expression possesses a versatile role in a myriad of cancers, but little is known about the role of LCN2 on osteosarcoma metastasis. In this study, we demonstrated that higher LCN2 inhibited cellular motility, migration, and invasion of osteosarcoma cells. Moreover, using RNA sequencing technology, we found that LCN2 repressed MET gene expression in U2OS cells. Manipulation of LCN2 levels influenced the migratory potential of osteosarcoma cells as cellular migration was enhanced by transfecting with vectors containing a constitutively active LCN2 cDNA and recombinant human LCN2. Moreover, the phosphorylation of mitogen-activated protein kinases/extracellular signal-regulated kinase (ERK) kinase (MEK) 1/2 and ERK 1/2 was decreased by LCN2 knockdown. Furthermore, the use of ERK inhibitor (U0126) and activator (tBHQ) confirmed that the pharmaceutic inhibition of MEK–ERK augmented the LCN2-mediated MET suppression and migration of U2OS and HOS cells. Conclusively, LCN2 inhibits osteosarcoma cell metastasis by suppressing MET via the MEK–ERK pathway.

## 1. Introduction

Cancer, associated with high mortality and disability rates, is one of the world’s largest health problems. Osteosarcoma, mainly arising from the metaphysis of long bones, is the most primary bone malignancy with a peak of incidence at 10–15 years and the second incidence peak in older adulthood [1,2]. Because the high rate of metastasis is a defining feature of osteosarcoma, its metastasis rate is responsible for the great majority of treatment failures and high mortality rates. Surgical en bloc resection or amputation of the diseased limb to achieve a complete radical excision had been the most common form of treatment for most osteosarcomas prior to metastasis [3], whereas 80% of patients had pulmonary metastasis (perhaps undetectable) at the time of presentation [4,5]. Recently, the combination of surgery and chemotherapy for osteosarcoma has increased long-term survival chances to approximately 68% through limb salvaging surgeries based on radiological staging, surgical techniques, and new chemotherapy protocols [3,6]. However, potent metastatic transfer to the lungs is still the main target of development of new therapeutic targets for anti-metastasis of osteosarcoma.

Cancer metastasis, the growth of primary tumor cells in a distant organ, includes cell migration, invasion, intravasation, traveling via circulation, extravasation, and eventual arrest at distant secondary sites [7]. In the invasion-metastasis cascade, mitogen-activated protein kinases (MAPKs), a family of serine/threonine kinases including extracellular signal-regulated kinase (ERK) 1/2, c-Jun N-terminal kinase (JNK) 1/2, and p38, are activated by MAPK/ERK kinase (MEK, MAPKK) to participate in the pathway progression of cell metastases [8]. The *MET* gene, first identified as an oncogene in the 1980s [9], encodes a transmembrane glycoprotein which possesses tyrosine kinase activity and its primary single chain precursor protein is post-translationally cleaved to produce the alpha and beta subunits to form the mature receptor for the hepatocyte growth factor (HGF) [10]. The MET receptor tyrosine kinase (c-Met) is highly expressed in more than 80% of osteosarcoma samples and its expression is correlated with high metastatic potential and a poor prognosis [11,12]. In osteosarcoma cells and preclinical models, the inhibition of c-Met effectively suppresses the metastatic phenotype [13].

Lipocalin 2 (LCN2), a prominent member of the lipocalin superfamily in human activated neutrophils, is originally identified as a 25-kDa secreted glycoprotein based on its covalent binding to the matrix metalloproteinase (MMP)-9 [14]. Higher LCN2 expression in cancerous cells compared with non-cancerous cells [15] and a pro-neoplastic role for LCN2 and related mechanisms have been demonstrated [16]. Since MMP-9 can degrade the extracellular matrix and basement membranes [17], LCN2 protects MMP-9 from degradation, thus increasing MMP-9 activity [18] to contribute to tumor progression and metastasis through formation of an LCN2/MMP complex [19]. Silencing of LCN2 represses invasion through a reduction in LCN2/MMP-9 complex formation in cholangiocarcinoma cells [20], which is linked to reduced survival of patients with cholangiocarcinoma of higher LCN2 expression [21]. However, LCN2 negatively modulates epithelial–mesenchymal transition (EMT) in hepatocellular carcinoma cells through the epidermal growth factor (EGF) or transforming growth factor (TGF)-β1/LCN2/Twist1 pathway [22]. In colon cancer cells, LCN2 may function as a metastasis suppressor [23]. Therefore, controversies related to the diverse effects of LCN2 remain, and especially in osteosarcoma are still unknown. Here, we tested the potential of LCN2 on human osteosarcoma metastasis and also investigated its underlying mechanisms.

## 2. Materials and Methods

### 2.1. Materials

Cell culture materials included Dulbecco’s modified Eagle medium (DMEM) and minimum essential medium (MEM) purchased from Gibco-BRL (Gaithersburg, MD, USA), and fetal bovine serum (FBS) purchased from Hyclone Laboratories, Inc. (South Logan, UT, USA). Propidium iodide (PI) and antibodies specific for p38, phosphorylated p38, and β-actin were obtained from BD Biosciences (San Jose, CA, USA). Additionally, antibodies specific for MET, MEK1/2, ERK1/2 and JNK1/2, and phosphorylated MEK1/2, ERK1/2, and JNK1/2 were purchased from Cell Signaling Technology (Danvers, MA, USA). Antibodies specific for LCN2 were purchased from R&D Systems (Minneapolis, MN, USA). Unless otherwise specified, all chemicals used in this study were purchased from Sigma-Aldrich (St. Louis, MO, USA).

### 2.2. Cell Culture

The human osteosarcoma HOS (13-yr-old female), MG-63 (14-yr-old male), and U2OS (15-yr-old female) cell lines were obtained from the Food Industry Research and Development Institute (Hsinchu, Taiwan), and the Saos-2 (11-yr-old Caucasian female) cell line was obtained from American Type Culture Collection (Manassas, VA, USA). They were supplemented with 10% FBS, 1% penicillin/streptomycin, and 5 mL of glutamine and cultured in DMEM and Eagle’s MEM media, respectively, and maintained at 37 °C in the humidified atmosphere of a 5% CO_2_ incubator as described elsewhere [24].

### 2.3. Protein Extraction and Western Blot Analysis

To investigate the molecular mechanism further, signaling pathways were detected by means of Western blot analysis. We plated 8 × 10^5^/dish HOS cells with or without overexpression of LCN2 and 6 × 10^5^/dish U2OS cells with or without knockdown of LCN2 on 6-cm cell culture dishes for 16 h. The total cell lysates of HOS and U2OS cells were prepared as described in previous studies [25], and Western blot analysis was performed using specific primary antibodies against LCN2, MEK1/2, or 3 MAPKs (ERK1/2, JNK1/2, and p38) with the specific antibodies for unphosphorylated or phosphorylated forms of the corresponding MEK1/2, ERK1/2, JNK1/2, or p38. As previously described, blots were then incubated with a horseradish peroxidase goat anti-rabbit or anti-mouse IgG for 1 h and the intensity of each band was measured via densitometry.

### 2.4. Transient Transfection

Cell transfection was carried out using LipofectAMINE 2000 (Invitrogen, Life Technologies, Carlsbad, CA) according to the manufacturer’s instructions. Vectors containing a constitutively active LCN2 cDNA (5 μg) and MET (5 μg) were diluted in 200 μL of MEM and then mixed with the transfection solution for 30 min as previously described [25]. Two days after transfection, the cells were used for the following experiment. Collected cells were processed for Western blot analysis, reverse transcription–polymerase chain reaction (RT–PCR), and cell migration assay.

### 2.5. Small Interfering RNA (siRNA)

To silence LCN2 protein expression, siRNA-inhibiting human LCN2 (s8114) and negative-control siRNA (4390844) were purchased from Applied Biosystems Instruments (Foster City, CA, USA). For the experiment, 6 × 10^5^/dish U2OS cells were grown on 6 cm cell culture dishes overnight. Following the manufacturer’s instructions, a total of 30 pM of LCN2 siRNA was transfected into the cells using lipofectamine RNAiMAX reagent (Invitrogen, Carlsbad, CA, USA). The silencer negative control siRNA, a nonsense siRNA duplex, was used as a control [24,26]. Similarly, a total of 50 pM of MET siRNA (s8700) was transfected into U2OS cells to silence MET protein expression as previously described.

### 2.6. Microculture Tetrazolium Colorimetric (MTT) Assay

For the cell viability experiment, we plated 8 × 10^4^/well HOS cells with or without overexpression of LCN2 and 8 × 10^4^/well U2OS cells with or without silencing of LCN2 in 24-well plates for 6 days, and a (3-(4,5-dimethylthiazol-2-yl)-2,5-diphenyltetrazolium bromide) (MTT) assay was performed to determine the cytotoxicity of LCN2 overexpression and knockdown. The media were removed and the HOS and U2OS cells were washed with phosphate-buffered saline. The media were subsequently changed and the cells were incubated with MTT (0.5 mg/mL) for 4 h as previously described [25].

### 2.7. Reverse Transcription–Polymerase Chain Reaction (RT–PCR)

For RT–PCR, we plated 8 × 10^5^/dish HOS cells and 6 × 10^5^/dish U2OS cells with or without silencing of LCN2 or MET, 4 × 10^5^/dish MG-63 cells, and 4 × 10^5^/dish Saos-2 cells on 6-cm culture dishes for 24 h. The RNA was entirely extracted using Total RNA Mini Kit (Geneaid, New Taipei City, Taiwan) and reverse transcribed into complementary DNA (cDNA) using a High Capacity cDNA Reverse Transcription Kit (Applied Biosystems, Foster City, CA, USA). Procedures of cDNA synthesis and the PCR amplification assay were performed as described in a previous study [24,25]. Specific primers were used for LCN2 and MET genes. For LCN2, the following forward (F) primers, and reverse (R) primers were used: F: 5′-TGATCCCAGCCCCACCT-3′, R: 5′-CCACTTCCCCTGGAATTGGT-3′. For SYBR-MET, the following forward (F) primers, and reverse (R) primers were used: F: 5′ATACggTCCTATggCTggTg3′, R: 5′TTgAgAggTTCTTTCCACCAAgT3′.

### 2.8. Flow Cytometry

To estimate the proportion of HOS and U2OS cells in different phases of cell cycle affected by transfecting with pcDNA vector for overexpression of LCN2 and siRNA directly against the LCN2 expression, cellular DNA contents and apoptosis were measured by flow cytometry as described elsewhere [27]. Using propidium iodide staining, cell cycle analysis by DNA content measurement employed flow cytometry to distinguish cells in different phases of the cell cycle.

### 2.9. Wound Healing Assay

To determine whether LCN2 alters the motility of HOS and U2OS cells, we plated 8 × 10^5^/well HOS cells with or without LCN2 overexpression and 6 × 10^5^/well U2OS cells with or without LCN2 knockdown on 6-well plates for 48 h and wounded them by scratching with a pipette tip. Afterwards, the cells were incubated with DMEM containing 0.5% FBS for 0, 4, 8, and 24 h for HOS cells and for 0, 6, 9, and 12 h for U2OS cells. Following previous studies, the cells were photographed using a phase-contrast microscope (×100) [26].

### 2.10. Cell Migration Assay

To examine the role of LCN2 on migration of U2OS and HOS cells in vitro, we employed a modified Boyden chamber without Matrigel coating. HOS cells with or without overexpression of LCN2 and U2OS cells with or without silencing of LCN2 were seeded into the upper section of the Boyden chamber (Neuro Probe, Cabin John, MD, USA) at densities of 2 × 10^5^/mL for HOS cells and 3 × 10^5^/mL for U2OS cells, and then incubated for 24 h at 37 °C. Subsequently, the cells of migration were counted under a light microscope [25].

### 2.11. MET Promoter-Driven Luciferase Reporter Assay

A density of 6 × 10^5^/dish U2OS cells with or without silencing of LCN2 were plated on 6-well plates for 24 h. The pGL3-basic vector, pGL3-control vector, and pGL3-MET promoter (−926 to +261) were co-transfected with a β-galactosidase expression vector (pCH110) as previously described. After 24 and 48 h of transfection, cell lysates were harvested, and luciferase activity was determined using a luciferase assay kit. The value of the luciferase activity was normalized to transfection efficiency and monitored by β-galactosidase expression [28].

### 2.12. Statistical Analysis

Statistical calculations of the data were performed using Student’s t-test for the differences between two independent samples and one-way analysis of variance (ANOVA) with post hoc Scheffe and Tukey’s tests for more than two groups with unequal and equal sample sizes per group, respectively. Each experiment was performed in triplicate and three independent experiments were performed. A *p*-value < 0.05 was considered statistically significant.

## 3. Results

### 3.1. Inconsistent LCN2 Expressions in Human Osteosarcoma HOS, MG-63, Saos-2, and U2OS Cells

**Figure 1:**
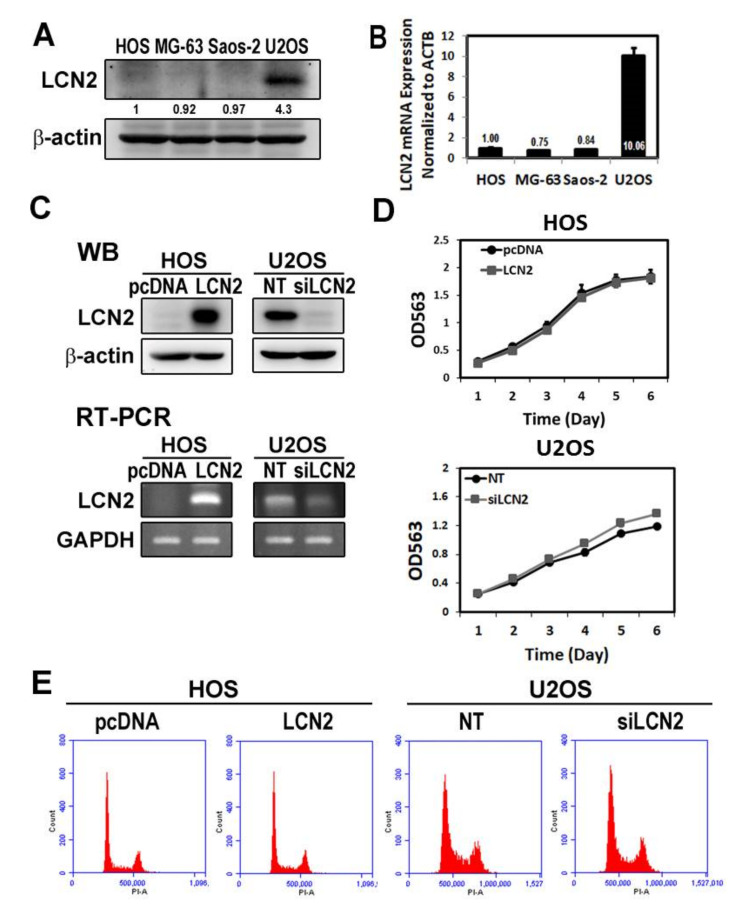
Effects of LCN2 on cell viability of human osteosarcoma cells. (**A**) Using Western blot analysis and (**B**) RT–PCR, the LCN2 protein and mRNA expressions in human osteosarcoma HOS, MG-63, Saos-2, and U2OS cells were detected, respectively. (**C**) Western blot analysis and RT–PCR for LCN2 protein and mRNA expressions of HOS cells after transfection with vectors containing a constitutively active LCN2 cDNA and U2OS cells after siRNA directly against the LCN2 expression were conducted. (**D**) Using an MTT assay, cell viability of HOS cells after transfection with vectors containing a constitutively active LCN2 cDNA and U2OS cells after siRNA directly against the LCN2 expression was detected up to 6 days and the effects are illustrated after quantitative analysis. (**E**) HOS cells after transfection with vectors containing a constitutively active LCN2, cDNA, and U2OS cells after siRNA directly against the LCN2 expression were subjected to flow cytometry after PI staining to analyze the cell cycle regulation.

To assess expressions of LCN2 in human osteosarcoma HOS, MG-63, Saos-2, and U2OS cells, Western blot analysis and RT–PCR were utilized. Our results expectedly showed inconsistent LCN2 mRNA and protein expressions in different cell lines: lower LCN2 mRNA and protein expressions were observed in HOS, MG-63, and Saos-2 cells; and the highest expressions were observed in U2OS cells, which showed about 10 times more of them in HOS, MG-63, and Saos-2 cells in RT–PCR (Figure 1A,B). Accordingly, we chose HOS and U2OS cells in all subsequent experiments to investigate the role of LCN2 in mechanisms underlying modulation of osteosarcoma metastasis.

### 3.2. Motility and Migration Are Inhibited in HOS Cells by Overexpression of LCN2 but Promoted in U2OS Cells by Silencing of LCN2

To determine whether LCN2 influences cellular motility, invasion, and migration, transfection with pcDNA vector for overexpression of LCN2 for HOS cells and siRNA directly against the LCN2 expression for U2OS cells was employed. We confirmed overexpression and knockdown of LCN2 protein and mRNA levels through Western blotting and RT–PCR in HOS and U2OS cells, respectively (Figure 1C). To demonstrate their cytotoxicity, MTT assay and flow cytometry showed that overexpression of LCN2 in HOS cells and silencing of LCN2 in U2OS cells up to 6 days did not increase the incidence of apoptosis, as evidenced by the absence of significant changes in the cell cycle (Figure 1D,E).

**Figure 2:**
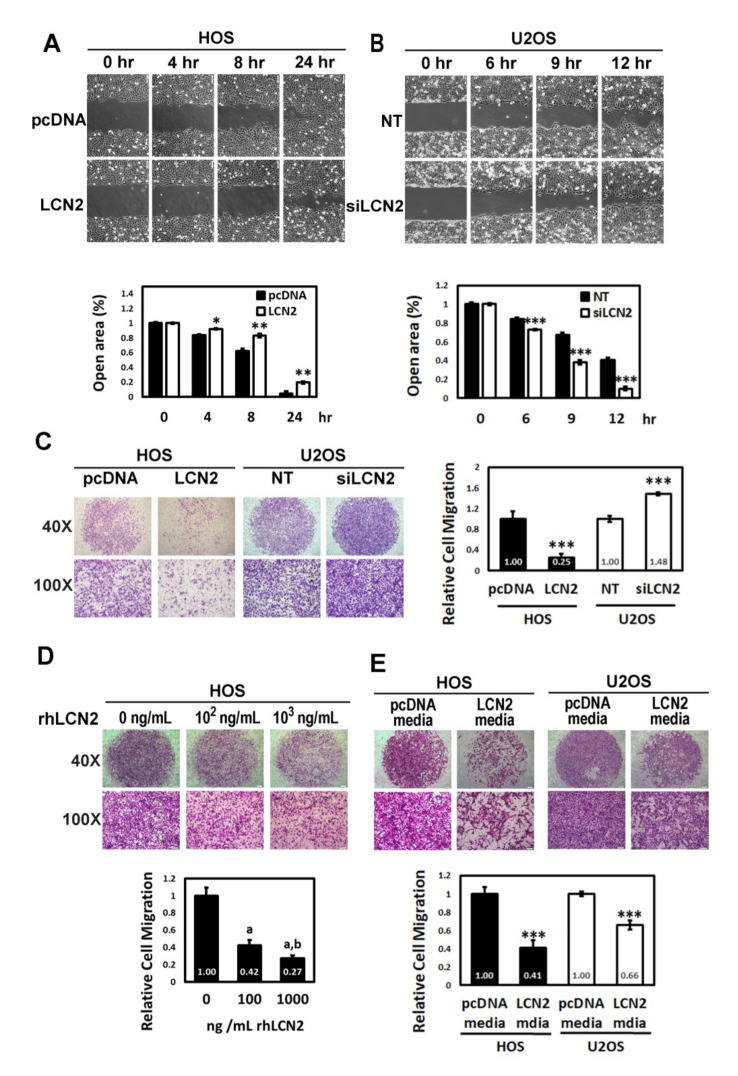
Effects of LCN2 on cell motility and migration of human osteosarcoma cells. (**A**,**B**) Effects of HOS cells after transfection with vectors containing constitutively active LCN2 cDNA and U2OS cells after siRNA directly against the LCN2 expression in wound healing assay were measured. * *p* < 0.05, ** *p* < 0.01, and *** *p* < 0.001 compared with the vehicle group. (**C**) Using a cell migration assay, migratory HOS cells after transfection with vectors containing a constitutively active LCN2 cDNA and U2OS cells after siRNA directly against the LCN2 expression were measured and subsequently subjected to quantitative analysis. *** *p* < 0.001 compared with the vehicle group. (**D**,**E**) Cell migration assays for HOS cells after treatment with various concentrations (0, 100, and 1000 ng/mL) of recombinant human LCN2 and both HOS and U2OS cells after treatment with culture media of LCN2 overexpression HOS cells were measured and subsequently subjected to quantitative analysis. F: 132.128, *p* < 0.001. ^a^ Significantly different, *p* < 0.05, when compared with control. ^b^ Significantly different, *p* < 0.05, when compared with 100 ng/mL. *** *p* < 0.001 compared with the vehicle group.

To verify the anti-metastatic actions of LCN2, we employed wound healing and modified Boyden chamber migration assays using HOS cells with or without overexpression of LCN2 and U2OS cells with or without silencing of LCN2 to compare cellular motility and migration. Intriguingly, overexpression of LCN2 significantly repressed motility and migratory potential in HOS cells, whereas LCN2 knockdown significantly enhanced those potentials in U2OS cells (wound healing: HOS: 4 h: *p* < 0.05, 8 h: *p* < 0.01, 24 h: *p* < 0.01; U2OS: 6 h: *p* < 0.001, 9 h: *p* < 0.001, 12 h: *p* < 0.001; migration: HOS: *p* < 0.001, U2OS: *p* < 0.001) (Figure 2A–C). To further test the potential effect of LCN2 expression on human osteosarcoma metastasis, the recombinant LCN2 protein and culture media of LCN2 overexpression HOS cells were used. By using recombinant human LCN2 in HOS cells, we confirmed that LCN2 dose-dependently suppressed the migratory response in the modified Boyden chamber migration assay (F: 132,128, *p* < 0.001) (Figure 2D). Culture media of LCN2 overexpression HOS cells significantly decreased the migratory potential of both HOS and U2OS cells, as expected (*p* < 0.001 and *p* < 0.001) (Figure 2E).

### 3.3. LCN2 Inhibits MET mRNA and Protein Expressions in HOS and U2OS Cells

**Figure 3:**
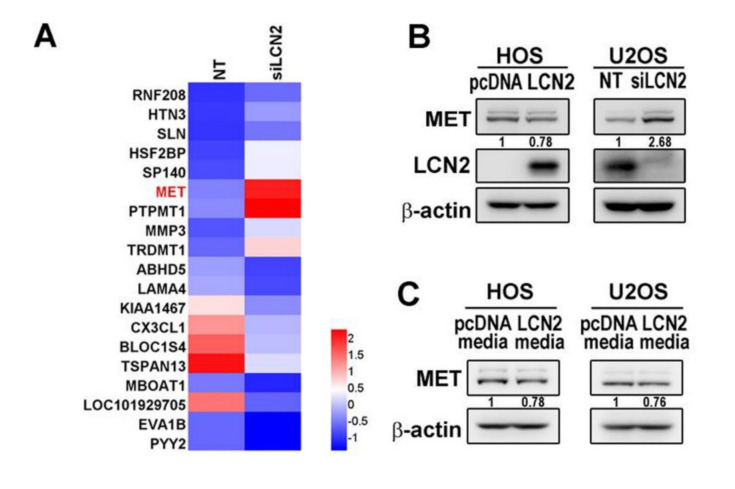

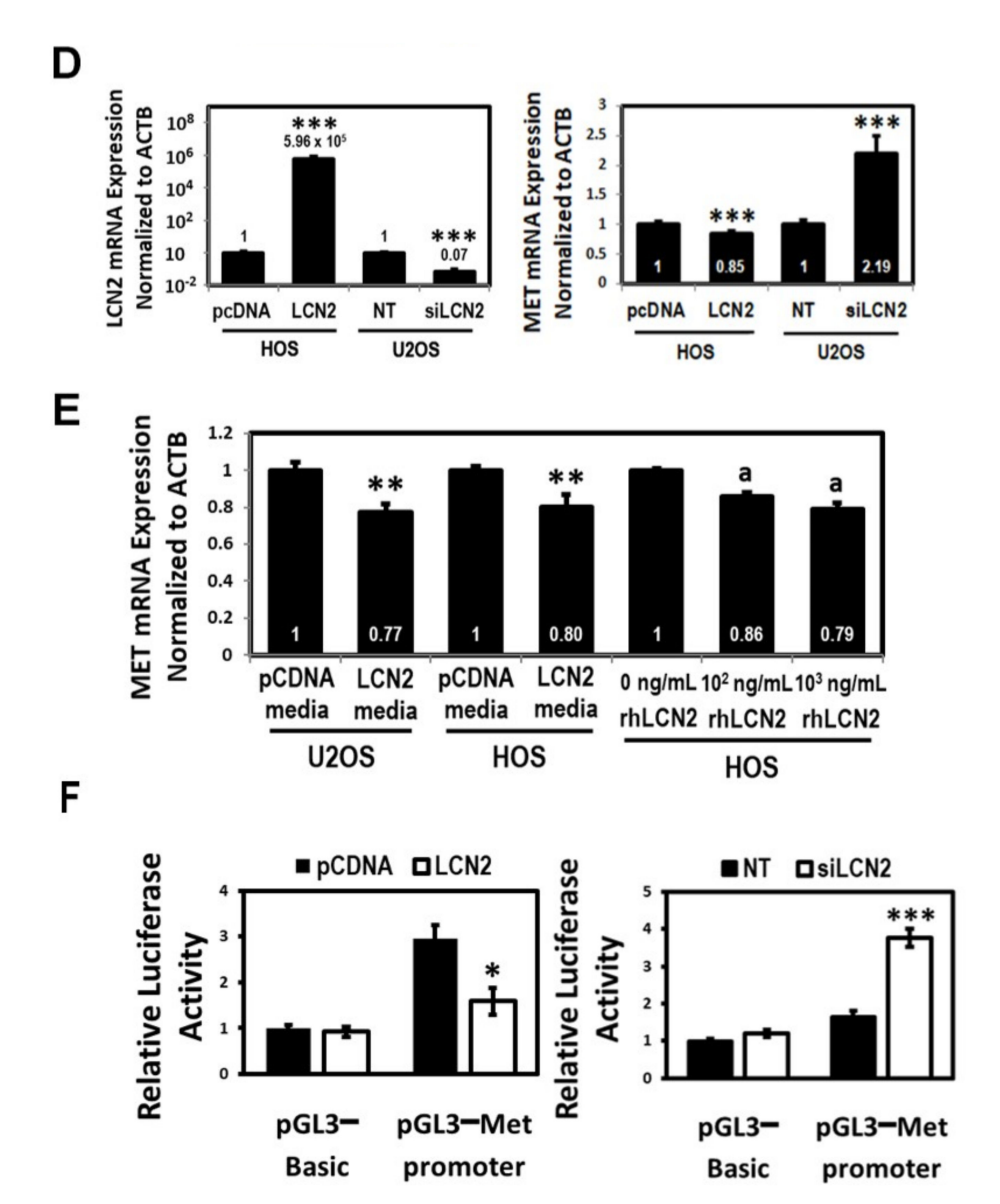
RNA sequencing for siLCN2 U2OS cells and effects of LCN2 on MET in HOS and U2OS cells. (**A**) Heat map of the hierarchical clustering of 19 differentially expressed genes identified in U2OS cells after siRNA directly against the LCN2 expression for 24 h. (**B**) Western blot analyses for the LCN2 and MET expressions in HOS cells after transfection with vectors containing a constitutively active LCN2 cDNA and U2OS cells after siRNA directly against the LCN2 expression were conducted. (**C**) Western blot analyses for the MET expression in both U2OS and HOS cells treated with culture media of LCN2 overexpression HOS cells were conducted. (**D**) RT–PCR for LCN2 and MET mRNA expressions in HOS cells after transfection with vectors containing a constitutively active LCN2 cDNA and U2OS cells after siRNA directly against the LCN2 expression were measured and subsequently subjected to quantitative analysis. *** *p* < 0.001 compared with the vehicle group. (**E**) RT–PCR for the MET mRNA expression in both U2OS and HOS cells treated with culture media of LCN2 overexpression HOS cells and HOS cells treated with various concentrations (0, 100, and 1000 ng/mL) of recombinant human LCN2 were measured and subsequently subjected to quantitative analysis. ** *p* < 0.01 compared with the vehicle group. F: 12,791, *p* < 0.01. ^a^ Significantly different, *p* < 0.05, when compared with control. (**F**) HOS cells were transfected with pcDNA vector for overexpression of LCN2 and U2OS cells were transfected with siRNA inhibiting human LCN2 for 24 h and then subjected to luciferase assay to analyze the promoter activity of MET. * *p* < 0.05, *** *p* < 0.001 compared with the vehicle group.

To explore the underlying mechanisms of the anti-metastatic actions of LCN2 in osteosarcoma cells, we conducted a transcriptomic analysis of U2OS cells transfected with siRNA inhibiting human LCN2 for 24 h using RNA sequencing technology. During the RNA sequencing analysis in siLCN2 U2OS cells, the expression levels of two upregulated DEG candidates, MET and PTPMT1, and two downregulated DEG candidates, BLOC1S4 and TSPAN13, were verified. Among them, MET is the most significantly upregulated gene in LCN2-knockdown cells and there are some reports regarding MET and osteosarcoma metastasis [11,12,13]. Furthermore, only one report regards promotion of human osteosarcoma cells progression by enhancing TSPAN13 expression [29]. Therefore, we chose the MET gene as the target gene to investigate its anti-metastatic properties. To validate the RNA sequencing findings of MET, we conducted Western blot analysis and interestingly found that HOS cells with LCN2 overexpression decreased the MET expression, while U2OS cells with LCN2 knockdown increased the MET expression (Figure 3B). Culture media of LCN2 overexpression of HOS cells expectedly suppressed the MET expression in both U2OS and HOS cells (Figure 3C). In RT–PCR, HOS cells treated with an overexpression of LCN2 significantly increased the LCN2 mRNA expression; U2OS cells treated with LCN2 knockdown significantly decreased the LCN2 mRNA expression (*p* < 0.001 and *p* < 0.001). Predictably, the MET mRNA expression was significantly decreased in HOS cells by overexpression of LCN2 but increased in U2OS cells by silencing of LCN2 (*p* < 0.001 and *p* < 0.001) (Figure 3D). In RT–PCR, both U2OS cells and HOS cells treated with culture media of LCN2 overexpression HOS cells significantly decreased the MET mRNA expression (*p* < 0.001 and *p* < 0.001), and recombinant human LCN2 also significantly down-regulated the MET mRNA expression in HOS cells (F: 12,791, *p* < 0.01) (Figure 3E). Collectively, the results implied that secreted glycoprotein LCN2 modulates expressions of the MET mRNA and protein.

We further identify whether LCN2 inhibits the promoter activity of MET to regulate the gene expression in U2OS cells. The luciferase reporter assay revealed that overexpression of LCN2 significantly decreased the promoter activity of MET in HOS cells while silence of LCN2 enhanced the promoter activity of MET in U2OS cells (Figure 3F), indicating the LCN2-induced inhibition of the mRNA of MET in U2OS cells at least partially at a transcriptional level.

### 3.4. MET Promotes U2OS and HOS Cells Migration

**Figure 4:**
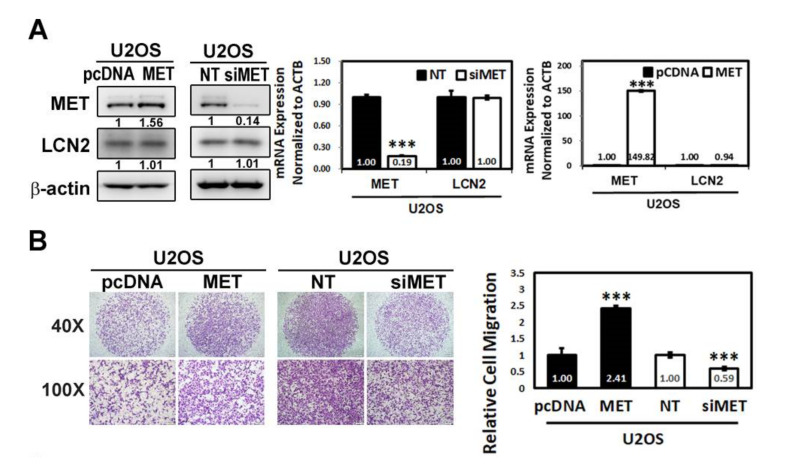

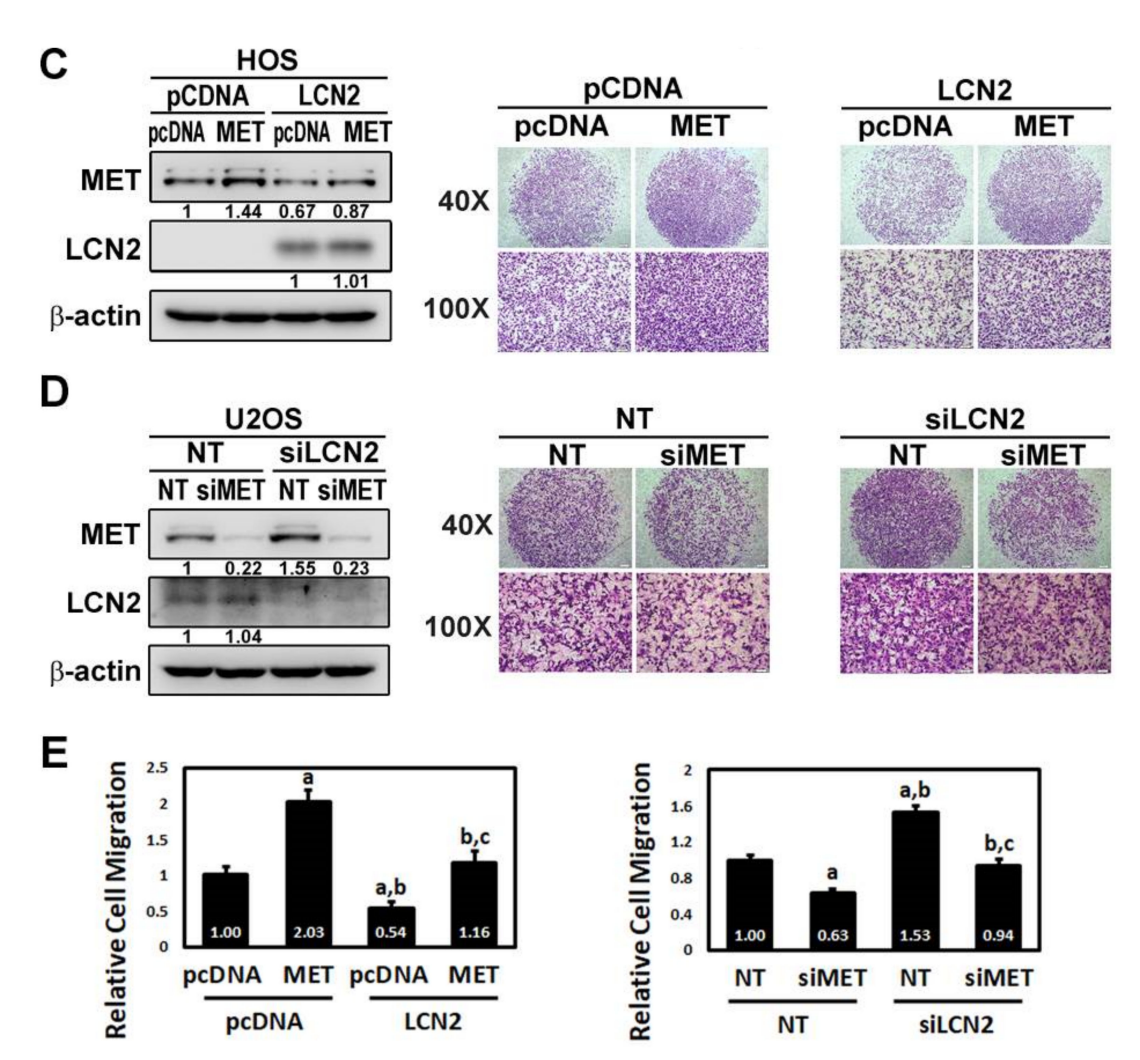
Effects of MET on U2OS and HOS cells migration. (**A**) Western blot analyses for MET and LCN2 proteins in U2OS cells after transfection with vectors containing a constitutively active MET cDNA and siRNA directly against the MET expression were measured. RT–PCR for MET and LCN2 mRNA expressions in U2OS cells after siRNA directly against the MET expression and U2OS cells after transfection with vectors containing a constitutively active MET cDNA were measured and subsequently subjected to quantitative analysis. *** *p* < 0.001 compared with the vehicle group. (**B**) Cell migration assays for U2OS cells after transfection with vectors containing a constitutively active MET cDNA and siRNA directly against the MET expression were measured and subsequently subjected to quantitative analysis. *** *p* < 0.001 compared with the vehicle group. (**C**–**E**) Western blot analyses and cell migration assays for HOS cells with or without overexpression of LCN2, transfected with vectors containing a constitutively active MET cDNA, and U2OS cells with or without knockdown of LCN2, siRNA directly against the MET expression, were measured, respectively, and migratory cells were subsequently subjected to quantitative analysis. HOS: F: 82.987, *p* < 0.001; U2OS: F: 150.955, *p* < 0.001. ^a^ Significantly different, *p* < 0.05, when compared with HOS cells without overexpression of LCN2 and MET, or U2OS cells without silencing of LCN2 and MET. ^b^ Significantly different, *p* < 0.05, when compared with HOS cells without overexpression of LCN2 but with overexpression of MET, or U2OS cells without silencing of LCN2, but with MET. ^c^ Significantly different, *p* < 0.05, when compared with HOS cells with overexpression of LCN2 but without overexpression of MET, or U2OS cells with silencing of LCN2, but without silencing of MET.

To validate whether MET is the downstream molecule of LCN2 and influences toward cell migration, U2OS cells transfected with vectors containing a constitutively active MET and siRNA directly against the MET expression were employed. Unsurprisingly, Western blot analysis showed that both overexpression and silencing of MET could not affect the LCN2 expression in U2OS cells (Figure 4A). In RT–PCR, MET knocked-down U2OS cells significantly decreased the MET mRNA expression and MET overexpression U2OS cells significantly increased the MET mRNA expression (*p* < 0.001 and *p* < 0.001), whereas both the MET knockdown and MET overexpression U2OS cells had no effect on LCN2 mRNA expression (*p* = 0.79 and *p* = 0.71), indicating that MET is not an upstream signal of LCN2. Moreover, the modified Boyden chamber migration assay showed that MET overexpression in U2OS cells significantly increased cellular migration and reversed the effect of MET silencing, significantly decreasing U2OS cellular migration (*p* < 0.001 and *p* < 0.001), (Figure 4B).

Transfection with vectors containing a constitutively active MET cDNA significantly increased migration of HOS cells without overexpression of LCN2 and reversed the decrease in migration of HOS cells with LCN2 overexpression, but had no effect on the LCN2 expression in HOS cells with or without LCN2 overexpression (F: 82,987, *p* < 0.001). In U2OS cells with or without knockdown of LCN2, transfection with siRNA directly against the MET expression significantly decreased migration of U2OS cells without knockdown of LCN2 and reversed the increase in migration in LCN2 knockdown U2OS cells, without affecting the LCN2 expression (F: 150,955, *p* < 0.001) (Figure 4C–E). Therefore, the results confirmed that MET is downstream signing of LCN2 to modulate migration of HOS and U2OS cells.

### 3.5. LCN2 Inhibits the MEK–ERK Pathway to Repress Migration of U2OS and HOS Cells

**Figure 5:**
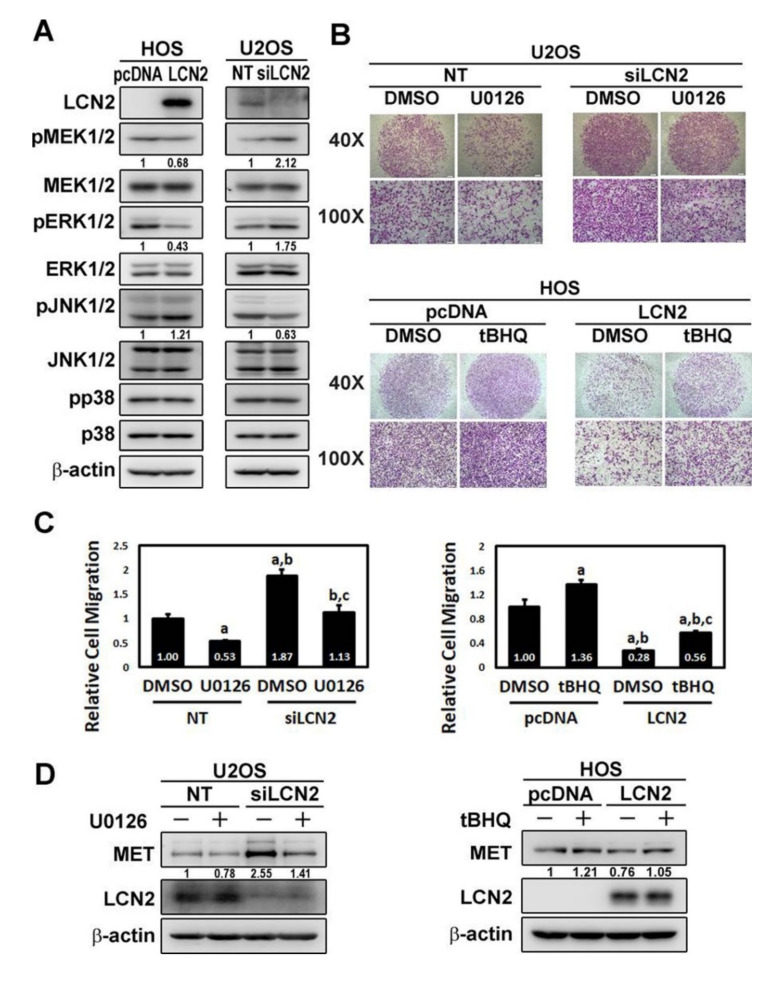
Effects of LCN2 on MEK and MAPKs to modulate MET and migration in U2OS and HOS cells. (**A**) Western blot analyses for HOS cells with or without overexpression of LCN2 and U2OS cells with or without knockdown of LCN2 to determine MEK and MAPKs, including their phosphorylation, were conducted, respectively. (**B**,**C**) Cell migration assays for effects of the inhibitor of ERK (U0126) on U2OS cells with or without silencing of LCN2 and the activator of ERK (tBHQ) on HOS cells with or without overexpression of LCN2 were measured and subsequently subjected to quantitative analysis. U2OS: F: 110,894, *p* < 0.001; HOS: F: 168,737, *p* < 0.001. ^a^ Significantly different, *p* < 0.05, when compared with U2OS cells without silencing of LCN2 and without U0126, or HOS cells without overexpression of LCN2 and without tBHQ. ^b^ Significantly different, *p* < 0.05, when compared with U2OS cells without silencing of LCN2 but with U0126, or HOS cells without overexpression of LCN2 but with tBHQ. ^c^ Significantly different, *p* < 0.05, when compared with U2OS cells with silencing of LCN2 but without U0126, or HOS cells with overexpression of LCN2 but without tBHQ. (**D**) Western blot analysis for U2OS cells with or without silencing of LCN2 and HOS cells with or without overexpression of LCN2 to determine effects of U0126 and tBHQ on MET and LCN2 expressions were conducted.

Since MEK and MAPKs pathways may be downstream signaling of LCN2, Western blot analysis was employed to further investigate its molecular mechanisms. In the analysis, MEK1/2 and MAPKs pathways were detected in HOS cells with or without overexpression of LCN2 and U2OS cells with or without knockdown of LCN2. As a result, LCN2 overexpression HOS cells decreased phosphorylation of MEK1/2 and ERK but LCN2 knockdown U2OS cells increased phosphorylation of MEK1/2 and ERK, indicating that LCN2 inhibits phosphorylation of MEK1/2 and ERK (Figure 5A). Additionally, increased phosphorylation of JNK1/2 in LCN2 overexpression HOS cells, decreased phosphorylation of JNK1/2 in LCN2 knockdown U2OS cells, and no obvious influence on p38 in both HOS and U2OS cells, including its phosphorylation, were observed. Based on these findings, the MEK–ERK cascade is suppressed by LCN2.

To further identify whether the inhibition of MEK–ERK phosphorylation by LCN2 interferes with the suppression of MET and cellular migration, we used the inhibitor of ERK (U0126) in U2OS cells with or without LCN2 knockdown and the activator of ERK (tBHQ) in HOS cells with or without LCN2 overexpression in Western blot analysis. As shown in Figure 5B,C, migratory potential of U2OS cells was activated by silencing of LCN2 (F: 110,894, *p* < 0.001). Conversely, migratory potential of HOS cells was repressed by overexpression of LCN2 (F: 168,737, *p* < 0.001). Interestingly, the inhibitor of ERK (U0126) significantly decreased the migratory response of U2OS cells with or without silencing of LCN2 (*p* < 0.05 and *p* < 0.05) and also significantly reduced the increase in migration in LCN2 knockdown U2OS cells (*p* < 0.05). Migratory potential of HOS cells with or without overexpression of LCN2 was significantly increased by the activator of ERK (tBHQ) (both *p* < 0.05). Expectedly, the activator of ERK (tBHQ) significantly reversed the decrease in migration of LCN2 overexpression HOS cells (*p* < 0.05). Taken together, these results provided better defining of the MEK–ERK signaling pathway which acts as downstream of LCN2.

### 3.6. LCN2-Inhibited Migration through Suppression of MET via the MEK–ERK Pathway in U2OS and HOS Cells

To further confirm whether the MEK–ERK pathway is localized at upstream of MET, Western blotting was subsequently performed. While the MET expression was predictably activated by silencing of LCN2 and reduced by the inhibitor of ERK (U0126) in U2OS cells with or without LCN2 knockdown, their LCN2 expressions could not be influenced by U0126. In HOS cells with or without overexpression of LCN2, the MET expression was repressed by LCN2 overexpression and enhanced by the activator of ERK (tBHQ), whereas tBHQ had no effect on LCN2 expressions (Figure 5D). Overall, these findings indicated that the MEK–ERK pathway plays a critical upstream role of MET in HOS and U2OS cells.

## 4. Discussion

As expected, we demonstrated inconsistent expressions of the cell-derived immune-related protein LCN2 in human osteosarcoma HOS, MG-63, Saos-2, and U2OS cells. Because the opposite effects of LCN on metastatic potential in two human colon cancer cell lines are very intriguing [23], we further explored the manipulation of LCN2 without cytotoxicity reducing cellular motility, invasiveness, and migratory potential in low and high LCN2 expressions of HOS and U2OS cells, respectively. Using RNA sequencing technology and the pathway enrichment analysis, the upregulated protein of MET was intriguingly observed after silencing of LCN2 in U2OS cells. To validate the RNA sequencing finding related to MET, we further confirmed that overexpression of LCN2 and recombinant human LCN2 in HOS cells, and culture media of LCN2 overexpression HOS cells in HOS and U2OS cells repressed mRNA and protein expressions of MET, whereas MET mRNA and protein expressions in U2OS cells were activated by silencing of LCN2. Nevertheless, both transfection with vectors containing a constitutively active MET cDNA and siRNA directly against the MET expression could not affect the LCN2 expression in HOS and U2OS cells. Migration of HOS cells with or without overexpression of LCN2 was induced by MET overexpression, whereas migration of U2OS cells with or without silencing of LCN2 was repressed by MET knockdown. Through a further analysis of upstream pathways of MET in LCN2-inhibited migration, we discovered that overexpression of LCN2 decreased phosphorylation of MEK1/2 and ERK1/2 in HOS cells, while silencing of LCN2 increased phosphorylation of MEK1/2 and ERK1/2 in U2OS cells. Notably, the inhibitor of ERK (U0126) attenuated the MET expression and migration in U2OS cells with or without LCN2 knockdown and the activator of ERK (tBHQ) promoted the MET expression and migration in HOS cells with or without LCN2 overexpression. These findings implied that LCN2′s anti-metastatic actions in human osteosarcoma HOS and U2OS cells resulted from attenuation of the MET expression and activity through the MEK–ERK pathway rather than JNK and p38 signaling.

In addition to some inflammation and pathological conditions [30,31], the versatile cytokine LCN2 has gained attention as a potential biomarker and a modulator of some human cancers, including breast [14], thyroid [32], colon [33], and pancreatic cancers [34]. In metastasis, LCN2 is initially identified as a promotor to induce EMT in breast cancer cells to enhance tumor invasion [35]. In prostate cancer cells, LCN2 plays an important role in facilitating cell migration and invasion of prostate cancer by inducing EMT through the ERK/SLUG axis [36], and the CXCL1-LCN2 axis triggers a cascade amplification event of Src signaling, EMT, and migration, leading to promotion of cancer metastasis [37]. However, disruption of the LCN2 gene in mice suppresses primary mammary tumor formation, whereas it does not decrease lung metastasis [38]. Thus, controversies over LCN2′s function remain in different cancer types.

Even in the same genetic origin, increasing LCN2 expression in poorly metastatic human colon cancer (KM12C) cells generates more invasive abilities while LCN2 short hairpin RNA has the opposite effect; nevertheless, over-expressing LCN2 in the highly metastatic human colon cancer (KM12SM) cell line greatly reduces its invasive behavior in vitro and in vivo [23]. Furthermore, LCN2 promotes EMT through Rac1, one of Ras homolog gene family (Rho) small guanosine triphosphate hydrolases (GTPases) and an integral regulator of EMT induction, to increase KM12C cell motility and invasion, indicating that LCN2 may be a target for therapeutic intervention in colorectal cancer cell metastasis [39]. Based on metastatic potential varied between the two cell lines used, the discrepancy between reports may be due to a difference in cell line specificity or to the fact that it is critical in advancing metastatic potential. Therefore, the role of LCN2 in anti-metastasis is remarkably divergent not only among the different cancer types but also between various cell lines in the same cancer. Using manipulation of LCN2 in low LCN2 expression of HOS cells and high LCN2 expression of U2OS cells, we confirmed LCN2 contributing to the anti-metastatic property in human osteosarcoma cells regardless of originally high or low expressions.

Generally, MET is thought to play a central role in signaling pathways to control cell proliferation, survival, and migration, in response to binding by its ligand HGF during developmental morphogenesis and in multiple cancer types [9,40]. In particular, expressions of MET/HGF receptor in osteosarcoma with and without bone metastasis were noted in 25 and 90% of cases, respectively [41]. However, we are intrigued that interactive crosstalk exists between MET and other members of the EGF receptor (EGFR) family, including human EGFR (HER)2 and HER3, and MET can be regulated through intermediary signaling pathways because of different requirements for MAPK signaling depending on different cell types, lines, and contexts [11,42]. For example, EGF-activated MET phosphorylation occurs through ERK and p38 pathways and compensation works via parallel MAPK cascades in mouse myeloid 32D cells [43]. A more complicated relationship presents in EGFR-MET crosstalk in non-small cell lung carcinoma cells of varying metastatic potential and EGFR modulates MET at multiple levels to enhance cellular migration and invasion, as demonstrated by our findings of a new tumor-specific crosstalk for the LCN2′s anti-metastatic action by attenuating MET through the MEK–ERK pathway in human osteosarcoma cells. To further investigate the inhibitory level of LCN2 on MET, we performed the luciferase reporter assay and observed that LCN2 knockdown U2OS cells significantly enhanced the MET promoter activity. Consequently, this indicates that downregulation of LCN2 on the MET expression in U2OS cells occurs at least partially at a transcriptional level.

Hence, we confirmed the novel discovery that illustrated the anti-metastatic property of LCNS involving the MEK–ERK pathway and downstream MET in human osteosarcoma. These data suggest a need for future research on anti-metastasis of LCN2 for osteosarcoma and render the molecular basis for better understanding of LCN2 as a novel anti-metastatic target for research of osteosarcoma in the future. Indeed, how it attenuates metastasis may be clear now and a pertinent concern is actually determining which molecules are involved in the MEK–ERK pathway when LCN2 inhibits the migratory ability of HOS and U2OS cells. Nevertheless, the limitations of this study include the lack of an in vivo study and the absence of information on how to manipulate LCN2 herein to translate it to clinical use; therefore, further studies are required to validate whether the anti-metastatic actions in vivo occurs as those in vitro.

## 5. Conclusions

Conclusively, human osteosarcoma HOS and U2OS cell-derived LCN2 contributes to inhibition of cellular migration through the MEK–ERK signaling pathway to downstream MET, and these findings imply an identified biomarker of LCN2 on anti-metastasis of osteosarcoma. The promotion of LCN2 expression to repress motility, invasion, and migration could be a potential target for anti-metastasis of osteosarcoma. This new discovery illustrates the involvement of the MEK–ERK pathway and downstream MET on migration of osteosarcoma, which in turn illustrates the pertinence of further studying LCN2 related to anti-metastasis for human osteosarcoma.

## Data Availability

The data presented in this study are available on request from the corresponding author.
